# Slowed Movement Stopping in Parkinson’s Disease and Focal Dystonia is Improved by Standard Treatment

**DOI:** 10.1038/s41598-019-55321-5

**Published:** 2019-12-20

**Authors:** Supriyo Choudhury, Akash Roy, Banashree Mondal, Ravi Singh, Saptak Halder, Koustav Chatterjee, Mark R. Baker, Hrishikesh Kumar, Stuart N. Baker

**Affiliations:** 1 Department of Neurology, Institute of Neurosciences Kolkata, Kolkata, India; 2 Department of Neurology, Royal Victoria Infirmary, Queen Victoria Rd, Newcastle upon Tyne, NE1 4LP United Kingdom; 3 Department of Clinical Neurophysiology, Royal Victoria Infirmary, Queen Victoria Rd, Newcastle upon Tyne, NE1 4LP United Kingdom; 4 Institute of Neuroscience, The Medical School, Newcastle University, Framlington Place, Newcastle upon Tyne, NE2 4HH United Kingdom; 5 Department of Physiology, University of Calcutta, Kolkata, India

**Keywords:** Motor control, Movement disorders

## Abstract

Patients with Parkinson’s disease and focal dystonia have difficulty in generating and preventing movement. Reaction time (RT) and stop signal reaction time (SSRT) measure the speed to initiate and stop a movement respectively. We developed a portable device to assess RT and SSRT. This incorporated a novel analysis to measure SSRT more efficiently (optimal combination SSRT, ocSSRT). After validation ocSSRT was measured in Parkinson’s disease patients without dyskinesia (PD), cervical dystonia (CD) and writer’s cramp. We also assessed how ocSSRT responded to L-dopa in PD patients and botulinum toxin injections in CD patients. Participants were instructed to release a button following a green LED flash on the device. On 25% of trials, a red LED flashed 5–195 ms after the green LED; participations were instructed to abort the button release on these trials. ocSSRT and RT were significantly prolonged in patients with Parkinson’s disease and focal dystonia (one-way ANOVA p < 0.001). Administration of L-dopa significantly improved ocSSRT and RT in PD patients (p < 0.001). Administration of botulinum toxin significantly improved ocSSRT, but not RT, in CD patients (p < 0.05). ocSSRT is an easily-administered bedside neuro-physiological tool; significantly prolonged ocSSRT is associated with PD and focal dystonia.

## Introduction

Patients with movement disorders have deficits in movement production but may also have abnormalities in preventing an undesired movement. Some patients with Parkinson’s disease (PD) have trouble terminating their gait sequence^1^, and deficient response inhibition has been reported in a number of other neurological disorders^2,3^.Whilst the neural substrate of the stopping process is largely unknown, structures such as the inferior frontal gyrus, pre-supplementary motor area, supplementary motor area and basal ganglia have all been implicated^4–7^.Reduced activity in the direct and cortico-subthalamic hyper-direct pathways in PD reportedly has a strong association with defective response inhibition^7–9^.

Investigation of movement inhibition was given a firm quantitative basis following the introduction of the stop signal paradigm by Logan and Cowan^10^. Subjects are asked to respond to a go cue, but to inhibit their responses on some trials if a stop cue appears. The paradigm is modelled as a race between a go and stop process which occur within the brain. Both processes are initiated by their respective cues, and race each other towards completion. If the go process finishes first, the response is executed; if the stop process finishes first, the response is successfully inhibited. The latency of this stopping process – the stop signal reaction time (SSRT) - is a covert expression of stopping ability and can be estimated using the race model. SSRT has been widely used in cognitive neuroscience, psychology and developmental neuroscience^11–13^. Patients with obsessive compulsive disorder and attention deficit hyperactivity disorder have prolonged SSRTs^11,14^, and SSRT is elevated in young children compared to young adults^15^. In movement disorders patients, SSRT was reported to be uncorrelated with the go signal reaction time in PD patients^2^. This suggests that the paradigm may measure stopping independently of any coincident bradykinesia.

Motor response inhibition has been previously reported to be deficient in patients with PD, focal hand dystonia and L-dopa induced dyskinesia compared to controls without neurological disorders^2,3,16,17^. Within the spectrum of PD patients, those with freezing of gait have impaired conflict resolution compared to those without this sign^18^. By contrast, SSRT is similar in PD patients with and without impulse control disorder^19^. Pharmacological studies in animals have revealed a role for dopamine and nor-adrenaline in modulating the stop signal response^20^, and consistent with this response inhibition is affected by dopamine receptor polymorphisms in healthy humans^21^. However, studies of PD patients in the ON and OFF states have so far shown conflicting results^19,22,23^.

In this study, we first introduce a new method to measure SSRT using portable equipment and an improved analytical approach. After validating this in healthy subjects, we then apply it to both PD and focal dystonia patients. We reveal not only that SSRT is prolonged in these conditions, but that it is improved by treatment. Differences between patients and healthy controls were sufficiently robust that measurement of SSRT may be of use in pre-screening for movement disorders.

## Methods

### Population and study procedure

The study was conducted in the movement disorders laboratory of a tertiary care referral centre in Eastern India. Ethical approval was obtained from the Institutional Ethics Committee of the Institute of Neurosciences, Kolkata; written informed consent was taken from all participants. All methods were performed in accordance with the relevant guidelines and regulation of the ethical approval.

The study was conducted in three parts.

**Experiment 1:** We measured optimal combination SSRT (ocSSRT) and average SSRT (avSSRT, see Supplementary Material for definitions) in 20 healthy subjects. Thereafter, the reliability of these measures was estimated by a repeat measurement in 14 subjects after a one-month interval; the remaining 6 healthy subjects were not available for retest. All healthy participants had no apparent neurological disease, were free from any uncontrolled systemic disease, and were not taking any neurotropic medications. None were colour blind by self-report and all had normal visual acuity (after correction if necessary). There was no history of substance abuse disorder or head injury.

**Experiment 2:** Measurements of SSRT were made in 30 patients with PD without dyskinesia, 20 patients with cervical dystonia (CD) and 10 patients with writer’s cramp (WC), to allow comparison with the healthy control data from Exp. 1. Patients with cognitive impairment (Mini Mental State Examination < 24), visual impairment or red-green colour blindness were excluded from the cohort^24^. PD patients were diagnosed using UK Brain Bank diagnostic criteria for PD and focal dystonia patients using the MDS consensus for dystonia^25,26^. The disease severity was assessed using standard disease severity scales: for PD, the Movement Disorders Society Unified Parkinson’s Disease Rating Scale-III (MDS UPDRS III)^27^ and Hoehn and Yahr (H&Y) scale^28^; for CD, the Toronto Western Spasmodic Torticollis Rating Scale (TWSTRS)^29^; for WC, the Writer’s cramp rating scale (WCRS)^30^. All assessments in PD patients were made in the OFF phase, without overnight medication.

**Experiment 3:** The effects of treatment on SSRT were measured. PD patients (n = 22) were requested to attend the clinic without overnight medication (OFF phase) for a baseline SSRT assessment. After this initial measurement, L-dopa was administered (according to the prescribed morning dose, range 100–300 mg, mean 167 mg,). The stop signal task was then re-evaluated within one hour (ON phase). For four of these patients, the OFF phase measurement used for this experiment was the same as that for Experiment 2. An additional nine patients who participated in experiment 2 returned on a separate day to participate in experiment 3, involving repeated measurement of SSRT in ON and OFF phase. Nine further patients participated who had not taken part in Experiment 3. In 10 CD patients, measures were made before and one month after a botulinum toxin injection in the affected muscles. All 10 patients had previously contributed to Experiment 2. The dose and the site of injections were selected by one of the authors (HK), who is an experienced movement disorders neurologist.

### Hardware

The hardware used to measure SSRT in this study comprised a battery-powered device housed within a plastic case, which the subject held comfortably in two hands (see Supplementary Fig. 1A). One red and one green LED (5 mm diameter) were positioned on the front of this box; beneath the LEDs was a press button (2 cm diameter). Above the LEDs was a four-line LCD screen, which provided a textual status display during the test. The device contained a dsPIC30F6012A microcontroller (Microchip Inc) programmed with custom firmware written in C using the MPLAB development environment. This controlled the task sequence, measured reaction times (1 ms precision) and response probabilities, and computed the results at the end. Numerical values for the estimated stop-signal reaction times were then displayed on the LCD screen and were copied down by the experimenter into the lab book. The device did not keep a lasting record of the single trial responses.

At the start of our study, some experiments were performed using similar physical hardware, connected to a laboratory interface (Micro1401, Cambridge Electronic Design, Cambridge, UK) and computer running Spike2 software (also Cambridge Electronic Design). The protocol was the same as for the microcontroller-based system, but all data were saved as Spike2 files, and subsequent analysis was carried out using scripts written in the Matlab environment (Mathworks Inc, Natick, USA).

### Test procedure

Participants sat comfortably in a semi-illuminated, quiet room holding the task device. The subject initiated a trial by pressing and holding down the response button. The LCD display then indicated the instruction ‘Release on green, hold on red’. After a delay (chosen from a uniform random distribution between 1 and 2.638 s), the green LED illuminated. On 75% of trials, no other LED illuminated, and the subject was required to release the button to respond (a GO trial). On 25% of the trials, the red LED illuminated, and the green LED extinguished after a delay (a NOGO trial); the subject was required not to release the button for correct performance. Four different stop-signal delays were used: 5, 65, 130 and 195 ms. Trials were presented in blocks of 32, with 24 GO trials and 8 NOGO trials (two for each delay) within a block. The order was determined at random but adjusted so that a NOGO trial was always preceded and followed by a GO trial. Following button release, there was a 1.3 s delay until the next trial started. During a NOGO trial, if the button was not released for 0.7 s after the green LED illuminated, this was considered a successful trial; the next trial started after a 2 s delay.

After two blocks of 32 trials, the task was paused for 60 s to allow the subject to rest. Subjects could also initiate a rest at any point by releasing the button, as the next trial did not start until the button was held down. Data gathering was stopped after three sets of 64 trials. Subjects were allowed to complete a few trials at the start to familiarise themselves with the task; these were discarded and not used for analysis.

### Statistical analysis

Mathematical details of our novel analytical approach are described in the Supplementary Materials. Analysis resulted in two measures – the average SSRT (avSSRT), which is measured as in previous studies, and the optimal combination SSRT (ocSSRT), which uses our novel approach. We also measured the median reaction time (RT). Across a population, categorical variables were described with percentage and numerical variables using mean (±SD). The unpaired multiple groups were compared using one-way ANOVA with post hoc Tukey’s test. The equality of variance between two unpaired groups was compared using Levene’s test. The criterion validity and test-retest reliability were assessed using the intra-class correlation coefficient between avSSRT and ocSSRT (considering avSSRT as the gold standard measure). The strength of meaningful correlation was estimated through the correlation coefficient and its level of significance. The homogeneity of variance was assessed between test-retest difference of avSSRT and ocSSRT through the Fligner-Killeen test (FK test). Cumulative distribution plots of SSRT across subjects were plotted, and from this receiver operating characteristic (ROC) curves constructed, which indicate whether a measure might have utility as a diagnostic tool. ROC curves were summarized by computing the area under the curve (AUC). An AUC of 0.5 indicates an identical distribution of the measure of interest in patients and controls; an AUC of 1.0 occurs if the distributions are entirely non-overlapping. Statistical analysis used the SPSS 20 statistical package (SPSS, Chicago, IL, USA) and custom-made MATLAB programs.

## Results

The baseline demographic and clinical characteristics of subjects are presented in Table 1.

**Table 1:** Demographic and disease characteristics of movement disorders patients and healthy controls.

	Parkinson’s Disease	Subset of Parkinson’s Disease patients who underwent assessment at OFF and ON phase	Cervical Dystonia	Writer’s Cramp	Healthy participants
Number	30	22	20	10	20
Male (%)	63%	64%	70%	100%	75%
Age in years (Mean ± SD)	59 ± 14.1**	66.5 ± 6.5**	43.3 ± 11.9 NS	53 ± 4.1 NS	37.4 ± 13.3
Disease Duration in years (Mean ± SD)	6.5 ± 4.3	6.2 ± 2.2	5.4 ± 2.3	3.2 ± 1.7	NA
Severity scale (Mean ± SD)	UPDRS III (OFF) 30.7 ± 10.6 H&Y (OFF) 3.2 ± 1.05	UPDRS III (OFF) 25.9 ± 6.8 H&Y (OFF) 2.4 ± 1.0	TWSTRS 14.1 ± 9.7	WCRS 5.29 ± 1.25	NA

The motor severity of Parkinson’s disease was estimated using Movement Disorders Society- Unified Parkinson’s Disease Rating Scale-III (MDS UPDRS III). The severity of cervical dystonia was estimated by Toronto Western Spasmodic Torticollis Rating Scale (TWSTRS) – pain and disability score. The severity of Writer’s cramp was estimated via the Writer’s cramp rating scale (WCRS) total score. Ages for the patient groups have been compared with the healthy participant cohort; **P < 0.001; NS, not significantly different (P > 0.05).

### Improved estimation of stop signal reaction time

**Figure 1:**
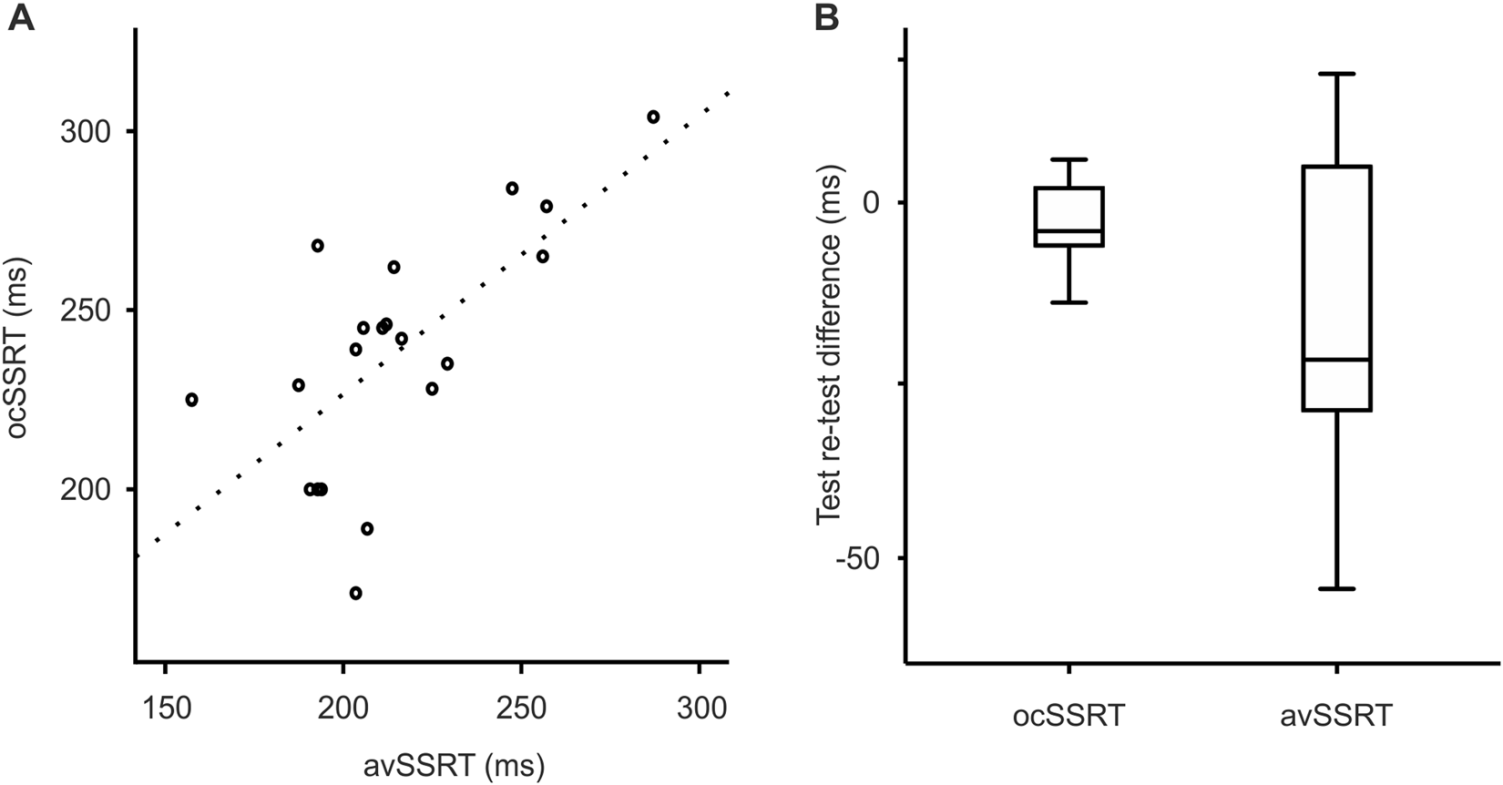
Validity and reliability of optimum combination stop signal reaction time (ocSSRT) compared to average stop signal reaction time (avSSRT). (**A**) Scatter plot showing association between avSSRT and ocSSRT. The intra-class correlation coefficient (ICC) of ocSSRT and avSSRT in 20 healthy volunteers was 0.79 (p = 0.001). The line of best fit (dotted line) shows a linear relationship between two measures. (**B**) Box and whisker plot compares the median difference and inter-quartile range (IQR, a measure of dispersion) of ocSSRT and avSSRT between two sets (baseline test and retest) of stop signal tasks (with an interval of one month) completed in 14 healthy individuals. The graph shows that test-retest difference of ocSSRT was significantly lower than avSSRT (Wilcoxon signed ranks test, p = 0.048). The dispersion of test-retest difference was significantly more for avSSRT (Fligner-Killeen test; p = 0.009). There is therefore an increased variability and reduced precision of avSSRT compared to ocSSRT.

We first compared the results of measuring SSRT using our new statistical approach (ocSSRT) with the more conventional procedure of simply averaging estimates made from trials with different stop signal delays (avSSRT). Figure 1A shows as a scatter plot the relation between these two measures in 20 healthy volunteers. In this case, for each person the two measures were made from the same recorded data. The intra-class correlation coefficient (ICC) between ocSSRT and avSSRT was 0.79 (significantly greater than zero, P = 0.001). However, it was noticeable that for some outliers, the two measures were quite different. For example, for one subject the avSSRT was 210 ms, whereas the ocSSRT was 171 ms. Further investigation revealed that this subject stopped his response perfectly for both the 5 ms and 65 ms stop signal delays, which led to an overestimate of SSRT at those delays. This inevitably produced a high value in the simple average (avSSRT). By contrast, the Bayesian approach naturally assigned high uncertainty to the measures at these delays, and gave little weight to them in the combination to generate ocSSRT. This illustrates the advantage of ocSSRT, which weights the different SSRT estimates by their reliability.

We then investigated the reliability of the two measures, by retesting 14 of the same subjects after one month. The ICC for avSSRT between the two repeats was 0.68, whereas it was 0.99 for ocSSRT (both significantly different from zero, p = 0.0001). Figure 1B presents the distribution of the difference in each parameter from the first to second measurement. The test-retest difference in the avSSRT was more variable than for ocSSRT (p = 0.009, FK test).

### Comparison of results in movement disorders patients with healthy controls

**Figure 2:**
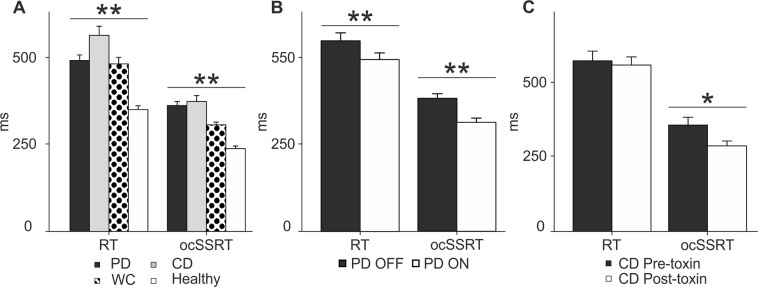
ocSSRT and reaction time (RT) in patients with movement disorders and after treatment. (**A**) ocSSRT and reaction time (RT) were significantly prolonged in patients with movement disorders compared to healthy participants. Overall differences among groups were compared by one-way ANOVA (p < 0.001). (**B**) ocSSRT and RT of patients with Parkinson’s disease reduce significantly from baseline (OFF state) to one hour after the administration of L-Dopa (ON state) (p < 0.001, paired sample t-test). (**C**) ocSSRT reduces significantly in patients with cervical dystonia from baseline to one-month post-treatment with botulinum toxin injection (p < 0.05, paired sample t-test).

Figure 2A presents the results for ocSSRT, and also the RT measured from the GO trials, for healthy controls and the various movement disorder patient groups. As might be expected, reaction times were significantly faster for the healthy controls than each patient group (controls, 350 ± 50 ms; PD OFF, 491 ± 88 ms; CD, 563 ± 118 ms; WC, 481 ± 60 ms; p = 0.0001 for comparison of each patient group with controls). In addition, the patients showed increased ocSSRT compared to the controls (controls, 238 ± 34 ms; PD OFF, 362 ± 62 ms; CD, 373 ± 76 ms; WC, 306 ± 25 ms; p = 0.0001, comparison of each patient group with controls). The standard deviation of ocSSRT was greater in CD but not in PD OFF and WC compared to healthy (controls, 34.1 ms; PD OFF, 62.1 ms; CD, 76.3 ms; WC, 25.3 ms; p = 0.07, 0.01, 0.43 respectively for PD, CD and WC). The extent of slowing of ocSSRT was not the same across the three movement disorders examined, with WC showing significantly shorter ocSSRT compared to PD (in OFF) and CD patients (p = 0.009 and p = 0.001 respectively). Notably, the RT (PD OFF 464 ± 52 ms, p < 0.001) and ocSSRT (PD OFF 368 ± 72 ms, p < 0.001) were also significantly prolonged in a subgroup of eight early PD patients (H&Y stage 1 and 2) compared to healthy controls. As expected, there were differences between this early subgroup and the remaining PD patients of our cohort in disease duration (4.8 ± 2.8 years vs. 8.4 ± 4.2 years, mean ± SD p = 0.03), UPDRS III score (20.7 ± 2.3 vs 30.4 ± 6.1, p = 0.005) and daily dose of levodopa (433 ± 44 ms vs 590 ± 105 mg, p = 0.001). The difference of RT and ocSSRT for PD patients persisted after adjustment for age as a covariant (p < 0.001).

In none of the patient groups investigated was there a significant correlation between RT and ocSSRT (Pearson’s correlation coefficient r: PD, r = −0.097, p = 0.61; CD, r = 0.137, p = 0.57; WC, r = −0.039, p = 0.94), although in healthy controls this correlation showed a non-significant trend (r = −0.426, p = 0.061).We observed no correlation with RT of TWSTRS (r = −0.379, p = 0.12), UPDRS III (r = −0.185, p = 0.42) or WCRS (r = −0.154, p = 0.74) in the respective patient groups. ocSSRT did not show a correlation with the severity of PD measured using either H&Y staging (r = −0.213, p = 0.26) or UPDRS III (r = 0.209, p = 0.42). Similarly, the severity of CD as estimated by TWSTRS was not correlated with ocSSRT (r = −0.03; p = 0.90).

### Improvement following standard treatment

Having demonstrated that these measurements are different in movement disorders patients, it was then of interest to determine whether they could be improved by standard treatments. Figure 2B shows a comparison of measures recorded in a sub-set of 22 PD patients in both the OFF and ON phase. RT was significantly decreased from 548 ms to 494 ms by taking L-dopa. ocSSRT was also significantly decreased, from 382 ms to 313 ms (both p = 0.0001). As might be expected, patients with the most prolonged ocSSRT in the OFF phase showed a trend for the greatest improvements from OFF to ON, although this just failed to reach significance (correlation coefficient between OFF ocSSRT and change in ocSSRT was 0.378, p = 0.083).By contrast, there was no relationship between the OFF RT and the change in ocSSRT from OFF to ON (correlation coefficient 0.117, p = 0.61).

One possible confounding factor in this result is that the PD patients were tested twice on the same day, which could have resulted in a learning effect. To check whether this was likely, we carried out further measurements in ten healthy subjects, who were retested twice on the same day with an interval between tests of just one hour. We found no significant differences between either RT (mean 358 ms *vs* 357 ms for first and second measurement respectively, p = 0.96) or ocSSRT (231 ms *vs* 230 ms, p = 0.95). We therefore conclude that changes seen in Parkinson’s patients were most likely due to an action of their dopaminergic medication, and not a learning effect caused by retesting on the same day.

Figure 2C compares measurements made in CD patients before, and one month after, their dystonia was treated by botulinum toxin injection. RT was unchanged by this treatment (before, 572 ms; after, 558 ms; p = 0.25), whereas there was a significant reduction in ocSSRT (before, 357 ms; after, 287 ms; p = 0.006).

### Separation of groups on the basis of response measures

**Figure 3:**
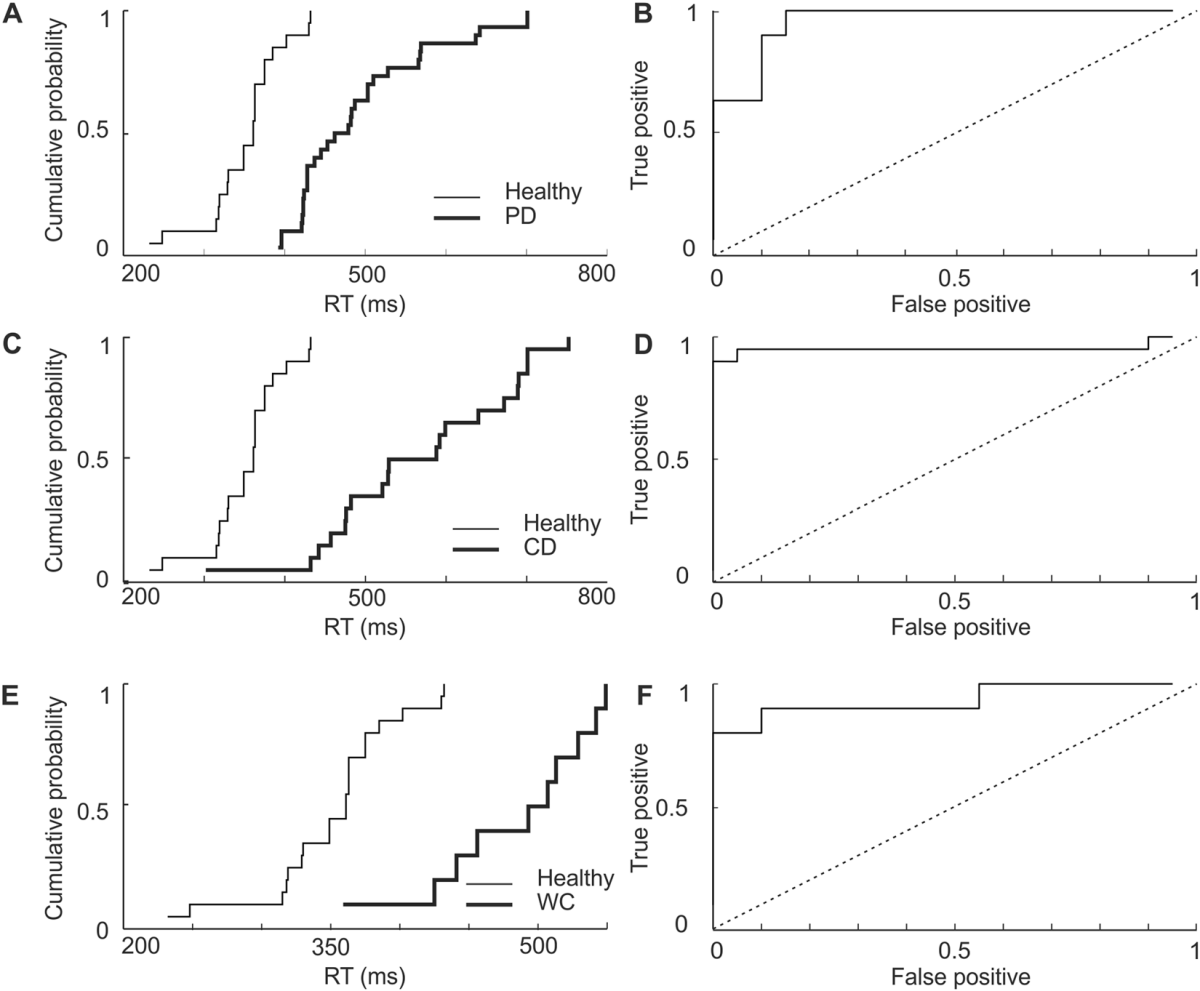
The cumulative probability and receiver operating characteristic (ROC) curves for RT in healthy controls and patients with movement disorders. (**A,C,E**) Cumulative probability distributions of RT in patients with Parkinson’s disease (PD), cervical dystonia (CD) and writer’s cramp (WC) (thick line) and healthy controls (thin line). (**B,D,F**) ROC plots derived from the cumulative probability distributions in (**A,C,E**) confirming that RT can effectively separate healthy controls from PD, CD and WC patients. Dotted diagonal line indicates expected result if RT did not discriminate the groups.

**Figure 4:**
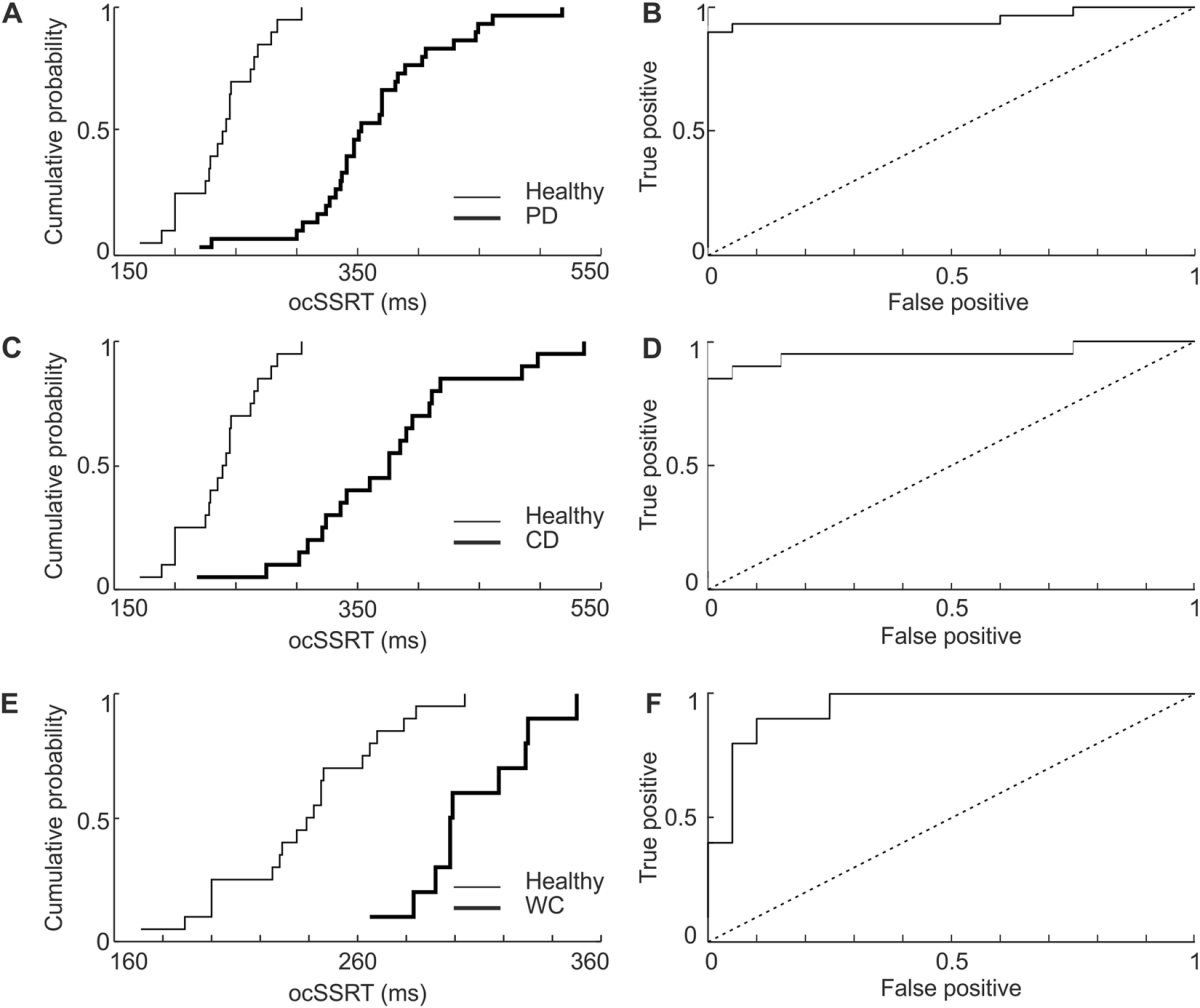
The cumulative probability and receiver operating characteristic (ROC) curves for ocSSRT in healthy controls and patients with movement disorders. (**A,C,E**) Cumulative probability distributions of ocSSRT in patients with Parkinson’s disease (PD), cervical dystonia (CD) and writer’s cramp (WC) (thick line) and healthy controls (thin line). (**B,D,F**) ROC plots derived from the cumulative probability distributions in (**A,C,E**) confirming that ocSSRT can effectively separate healthy controls from PD, CD and WC patients. Dotted diagonal line indicates expected result if ocSSRT did not discriminate the groups.

Figure 2A revealed a significant difference of RT and ocSSRT between movement disorders patients and healthy controls at a population level. It is of interest to know whether this difference was sufficiently robust to allow reliable diagnosis of individuals. This is explored in Figs. 3 and 4, which presents cumulative probability distribution plots (Figs. 3A,C,E and 4A,C,E) for each movement disorder group in comparison to healthy controls. These are then used to compile the corresponding receiver operating characteristic (ROC) curves (Figs. 3B,D,F and 4B,D,F). For PD, CD and WC patients, there was a clear separation in the distribution of RT and ocSSRT from healthy controls. This led to ROC curves which lay far from the identity line (dotted in Figs. 3B,D,F and 4B,D,F). The area under the ROC curve for ocSSRT was 0.903, 0.903 and 0.898 for PD, CD and WC patients respectively. For comparison, the area under the ROC curve for RT was 0.908, 0.904 and 0.885 and for avSSRT (data not illustrated) was 0.947, 0.925 and 0.935 for PD, CD and WC patients respectively. All of these values were significantly higher than 0.5 which would be expected for entirely overlapping distributions (p = 0.0001, Monte Carlo test).

As noted above, although WC patients had a significantly slower ocSSRT than healthy subjects, the extent of this slowing was smaller than for either PD or CD groups. ROC analysis showed that WC patients could be significantly discriminated from PD and CD patients on the basis of ocSSRT (area under ROC curve 0.825 and 0.85 respectively; both significantly different from 0.5, p = 0.0001, Monte Carlo test). By contrast, WC patients could not be significantly discriminated from PD or CD patients on the basis of RT (area under the curve 0.650 and 0.713) or avSSRT (area under the curve 0.668 and 0.690).

## Discussion

### Optimal combination SSRT as a measure of the stopping process

In this study we introduced ocSSRT as an improved method for estimating SSRT. Most of the literature has reported results based on the avSSRT. The ocSSRT was in agreement with the avSSRT when measured from the same experiment, thus validating this newly-developed statistical measure. However, the test-retest reliability for a group of healthy participants who performed the trial on two separate days over an interval of one month showed a much stronger agreement between ocSSRT compared with avSSRT. Either SSRT measure could therefore be used interchangeably in cross-sectional studies where the aim is to compare stopping process among various groups, although the decreased variability of ocSSRT will increase the statistical power of comparisons and there would be more chance for the differences to reach significance with fewer subjects. For a prospective study, ocSSRT would also have advantages over avSSRT due to its higher test-retest reliability.

### Prolonged SSRT in PD patients

In our study we observed that both RT and ocSSRT were significantly prolonged in PD compared to healthy volunteers. Interestingly, in healthy controls RT and ocSSRT showed a trend towards positive correlation, whereas these measures were uncorrelated in PD. In agreement with our findings, Gauggel *et al*. compared the stop signal task in 32 patients with PD and 31 orthopaedic controls^2^. The extent of bradykinesia was unrelated to SSRT in PD, but in the orthopaedic controls initiation speed could explain some of the variance in time taken to inhibit action. It is likely that some common processes influence both response speed and stopping, and that these vary across healthy individuals to generate a weak correlation. However, the pathological processes which lead to slowing in RT and SSRT appear separable.

### Prolonged SSRT in focal dystonia patients

We also observed prolonged SSRT in CD and WC patients compared to healthy participants. The result in WC is in agreement with previous work, which demonstrated a lower rate of response inhibition in patients with task specific focal hand dystonia^3^. It is intriguing that we additionally found elevated SSRT in CD, as our task involved releasing a button with the hand as a response; the hand was not affected by the dystonia in these individuals. The size of the increase in SSRT was actually larger in CD than in WC (Fig. 3A), emphasising that congruence between the muscles used to respond in the task and those involved in the dystonia was not an important factor. Even in WC, our results were surprising as the response movement required by the task did not induce dystonia, which was specific to writing. It seems likely that these patients have a more general underlying pathology in networks for response inhibition, which is unrelated to the nature of the focal presentation of the dystonia itself.

One factor to be considered is that both SSRT and RT might be affected by cognitive decline. In this context our findings in focal dystonia are interesting, since these disorders do not have a cognitive component, unlike PD. In addition, SSRT and RT changes were seen in early stage PD patients, where cognitive decline should be low. It seems therefore that the rises in SSRT and RT reflect, at least in part, a genuine component of the movement disorder, rather than being simply related to cognitive changes.

### SSRT is not a predictor of disease severity

Although all groups of movement disorders patients tested showed prolonged SSRT, within a particular disease group there was no correlation between SSRT and clinical measures of disease severity. Previous studies also failed to find a relation between the prolonged SSRT of PD patients and global measures of cognitive impairment or severity of disease^2,31^. By contrast, a more specific assessment of the severity of levodopa-induced dyskinesia in PD patients does show a correlation with SSRT^32^. The disease severity demonstrated no significant correlation with RT. This is expected as the major deficit in PD is bradykinesia and not the prolongation of reaction time^9^. It is likely that a multitude of changes to cortical and sub-cortical networks result from disease pathology and attempts at compensation to restore function. Global disease severity probably results from a myriad of different interactions and impairments, with many possible routes to generate a similar level of impairment. Only a sub-set of these networks will affect response inhibition as measured by SSRT; these may overlap with the circuits responsible for dyskinesia^32^. Interestingly, the ocSSRT was significantly prolonged even in a sub-set of patients with early stage of disease, suggesting that ocSSRT might be developed as an early disease marker.

### Standard treatments partially normalise SSRT

Although SSRT did not correlate with disease severity, when patients were treated SSRT was reduced. In PD patients, the SSRT was significantly reduced by levodopa treatment. This is in agreement with past work which showed an improvement in response inhibition in the ON phase^22^, although two previous studies failed to find a decrease specifically in SSRT with dopaminergic medications^19,23^. It is possible that our improved approach to estimation of SSRT allowed us to detect a relation, which could otherwise be masked by measurement variability.

It is perhaps not unexpected that treatment with a centrally-acting drug such as levodopa could modulate response inhibition. Interestingly, the patients with more prolonged OFF ocSSRT (unlike with more prolonged OFF RT) demonstrated a positive correlation with change in ocSSRT after levodopa administration. This differential correlation could suggest that RT and ocSSRT may be measuring underlying processes which are differently affected by the disease.

More unexpected was our finding that treatment of dystonia with botulinum toxin injections into the dystonic muscles also reduced SSRT. A direct central effect of the toxin is unlikely, as we expect minimal passage across the blood brain barrier. It is possible that the reduced SSRT is simply a consequence of the reduction of symptoms. Abnormal movements or posturing generated by CD could have engaged the patient in compensatory movements, thereby diverting attention from the stop signal task during the pre-treatment assessment. After effectively treating the dystonic condition with botulinum toxin, the cause of this inattention would be removed, allowing the patient to perform the task without diversion. However, against this explanation, the reaction time was not improved by botulinum toxin injection; this should be affected similarly to SSRT if the underlying cause is simply a reduction in distracting dystonia. This might suggest that RT and ocSSRT measure different underlying processes which respond to therapy in a different way. We suggest that the most likely cause for the observed SSRT reduction is that toxin therapy produced a chronic alteration of sensory input to the brain, which modulated the abnormal central network. In support of this idea, the long latency trans-cortical reflex in idiopathic focal dystonia is reduced significantly following botulinum toxin therapy^33^, and cortico-spinal plasticity is changed^34^.

## Conclusions

In this study, we have developed and validated an improved measure of SSRT and shown that implementing this in a portable device can deliver a simple and reliable test suitable for clinical use. Measures in patients were not just significantly different from healthy controls at a group level, but also showed sufficiently robust differences to allow accurate separation of individuals. Furthermore, WC patients could be separated from PD and CD patients only on the basis of our improved ocSSRT measure. The sub-set of patients with early symptoms of PD also demonstrated an abnormally prolonged ocSSRT. This suggests that SSRT could find utility as a marker for enhanced diagnosis (e.g. rapid throughput pre-screening) and to assess the response to treatments.

## Supplementary information

Supplementary information 
