# Supplementary material for: Slowed Movement Stopping in Parkinson’s Disease and Focal Dystonia is Improved by Standard Treatment
Source: Sci Rep. 2019 Dec 20;9:19504. doi: 10.1038/s41598-019-55321-5 (PMC6925208; doi:10.1038/s41598-019-55321-5)
Supplement: Supplementary file 1 — Supplementary information [file 41598_2019_55321_MOESM1_ESM.docx]

**Slowed Movement Stopping in Parkinson’s Disease and Focal Dystonia is Improved by Standard Treatment**

**Supriyo Choudhury^1†^, Akash Roy^1†5^,**Banashree Mondal^1^, Ravi Singh^1^, Saptak Halder^1^, Koustav Chatterjee^1^, Mark R. Baker^2, 3, 4^, **Hrishikesh Kumar^1^, Stuart N. Baker^4^**

**^†^SC and AR have equal contribution to this study.**

1. Department of Neurology, Institute of Neurosciences Kolkata, Kolkata, India

2. Department of Neurology, Royal Victoria Infirmary, Queen Victoria Rd, Newcastle upon Tyne, NE1 4LP, United Kingdom

3. Department of Clinical Neurophysiology, Royal Victoria Infirmary, Queen Victoria Rd, Newcastle upon Tyne, NE1 4LP, United Kingdom

4. Institute of Neuroscience, The Medical School, Newcastle University, Framlington Place, Newcastle upon Tyne, NE2 4HH, United Kingdom

5. Department of Physiology, University of Calcutta, Kolkata, India

a) **Name, address, telephone and email address of the joint corresponding authors:**

Prof. Stuart N Baker

Address:

Institute of Neuroscience

Henry Wellcome Building

The Medical School

Framlington Place

Newcastle University

Newcastle upon Tyne

NE2 4HH

UK

Email: [stuart.baker@ncl.ac.uk](mailto:stuart.baker@ncl.ac.uk)

Telephone: +44 (0) 191 208 8206

Fax: +44 (0) 191 208 5227

Dr Hrishikesh Kumar

Department of Neurology,

Institute of Neurosciences, Kolkata

185/1 AJC Bose Road, Kolkata

West Bengal (India)

Postal code: 700017

Email address: [rishi_medicine@yahoo.com](mailto:rishi_medicine@yahoo.com)

Phone number: +91 33 40309982

Fax number: +91 33 22867097

# Supplementary Material

## Data Analysis

The data recorded during task performance comprised a list of reaction times for a GO trial, *t_i_* , where *i*=1..*n* (*n* is the number of GO trials). These reaction times were sorted into order, so that *t_i_*≤*t_i+1_*. If the subject failed to respond on a GO trial within 700ms, the reaction time was recorded on that trial as 700ms. For the NOGO trials, we record the number of times that the subject incorrectly responded by a button release as M*_j_*, where j=1..4 indexes the four stop-signal delays *D_j_*=[5,65,130,195ms], out of a total of *N_j_* trials where this stop-signal delay was delivered.

Based on the race model of Logan and Cowan^35^, we can form a simple estimate of the stop-signal reaction time *SSRT_j_* from the trials where the stop-signal delay was *D_j_* as follows. We assume that the subject responded inappropriately on a fraction q*_j,_* of trials, estimated as q*_j_*=*M_j_*/*N_j_*, because on this proportion of reaction times the response occurred too early to be stopped:

$t<{SSRT}_{j}+D_{j}$ (Eq. 1)

Recalling that the reaction times *t_i_* are ordered from lowest to highest, computing the SSRT is a simple matter of accessing the relevant point of the reaction time distribution:

${SSRT}_{j}=t_{i}-D_{j} where i=q_{j}n$ (Eq. 2)

This yields four separate estimates *SSRT_j_*. Following Logan and Cowan^35^, the easiest way to combine these into a single estimate is to form the average SSRT (avSSRT):

$avSSRT=\frac{1}{4}\sum_{j=1}^{4} {SSRT}_{j}$ (Eq. 3)

This approach follows previous work and has the merit of simplicity, but it does not take into account uncertainties in the experimental observations. In practice, because the great majority (75%) of trials are GO trials, the distribution of reaction times *t_i_* will be well characterised. Supplementary Figure 1B illustrates an example experimental histogram. When transformed to an estimated cumulative probability distribution (Supplementary Figure 1C), the curve is very smooth. Assuming that the experimental observations accurately mimic the underlying distribution will thus introduce only small errors. Far greater errors might be introduced by the assumption that the proportion of NOGO trials on which responses are generated *q_j_* is equal to the observed fraction *M_j_*/*N_j_*, since *M_j_* could be quite small. For the example dataset shown in Supplementary Figure 1D, at the shortest stop-signal delay there were only 3 responses out of 16 trials. We estimate *q_1_*=3/16=0.1875, but this estimate would have been altered by 33% if the subject had responded differently on just one trial. Intuitively, an estimate of SSRT made from this stop-signal delay will be much less accurate than one where there are more responses; however Eq. 3 weights all estimates equally.

We take a Bayesian approach to this problem. Assume that for a given stop-signal delay *D_j_*, the actual SSRT is *SSRT_j_*. Based on Eq. 2, this implies a unique value of *q_j_*, the expected proportion of NOGO trials on which a response will be generated. During the experiment, we observed a particular sequence of *M_j_* trials with responses and *N_j_-M_j_* trials with no responses. The likelihood of seeing this experimental observation is:

$P\left( M_{j}responses out of N_{j} \right|q_{j})={q_{j}}^{M_{j}}{(1-q_{j})}^{N_{j}-M_{j}}$ (Eq. 4)

Based on Bayes’ rule

$P\left( q_{j} | M_{j}responses out of N_{j} \right)=\frac{P\left( M_{j}responses out of N_{j} | q_{j} \right)P(q_{j})}{P(M_{j}responses out of N_{j})}$ (Eq. 5)

*P*(*q_j_*) and *P*(*M_j_ responses out of N_j_*) are priors within a Bayesian framework. Since we have no expectations of either, we set these to be uniform (flat) priors, so that:

$P\left( q_{j} | M_{j}responses out of N_{j} \right)\propto P\left( M_{j}responses out of N_{j} | q_{j} \right)$ (Eq. 6)

This relates the likelihood of a particular response rate *q_j_* if we have observed *M_j_*/*N_j_* responses, to the probability of making the experimental observation of *M_j_* responses from *N_j_* if the underlying response rate is *q_j_*. From Eq. 2, there is a one-to-one relation between *q_j_* and *SSRT_j_*, hence Eq. 6 can be rewritten replacing *q_j_* with *SSRT_j_*. It only remains to determine the constant of proportionality; this is straightforward, using the fact that the integral of a probability distribution must be one. We can therefore find *P*(*SSRT_j_* | *M_j_ responses out of N_j_*), the likelihood of a particular value of SSRT given our experimental observation. These four probability curves are shown in Fig. 1E for the illustrated dataset.

The various SSRT likelihoods arising from the different stop-signal delays can be simply combined by multiplication, yielding one estimate of the SSRT probability distribution:

$$P\left( SSRT|Experimental data \right)\propto\prod_{j=1}^{4} P({SSRT}_{j}|M_{j}responses out of N_{j})$$

This combined estimate is shown in Fig. 1F. Note that this distribution is narrower than any of the component distributions, reflecting the increased certainty in the SSRT value arising from including more experimental data. Once we have the complete probability distribution, it is straightforward to determine its mean, as the optimal combination SSRT (ocSSRT):

$$ocSSRT=\int_{0}^{\infty} SSRT P\left( SSRT \right)dSSRT$$

This is illustrated as the vertical line on Supplementary Fig. 1F. Furthermore, we can find the 2.5% and 97.5% points of the distribution, which will estimate the 95% confidence limits on the SSRT (shown with grey shading in Supplementary Figure 1F).


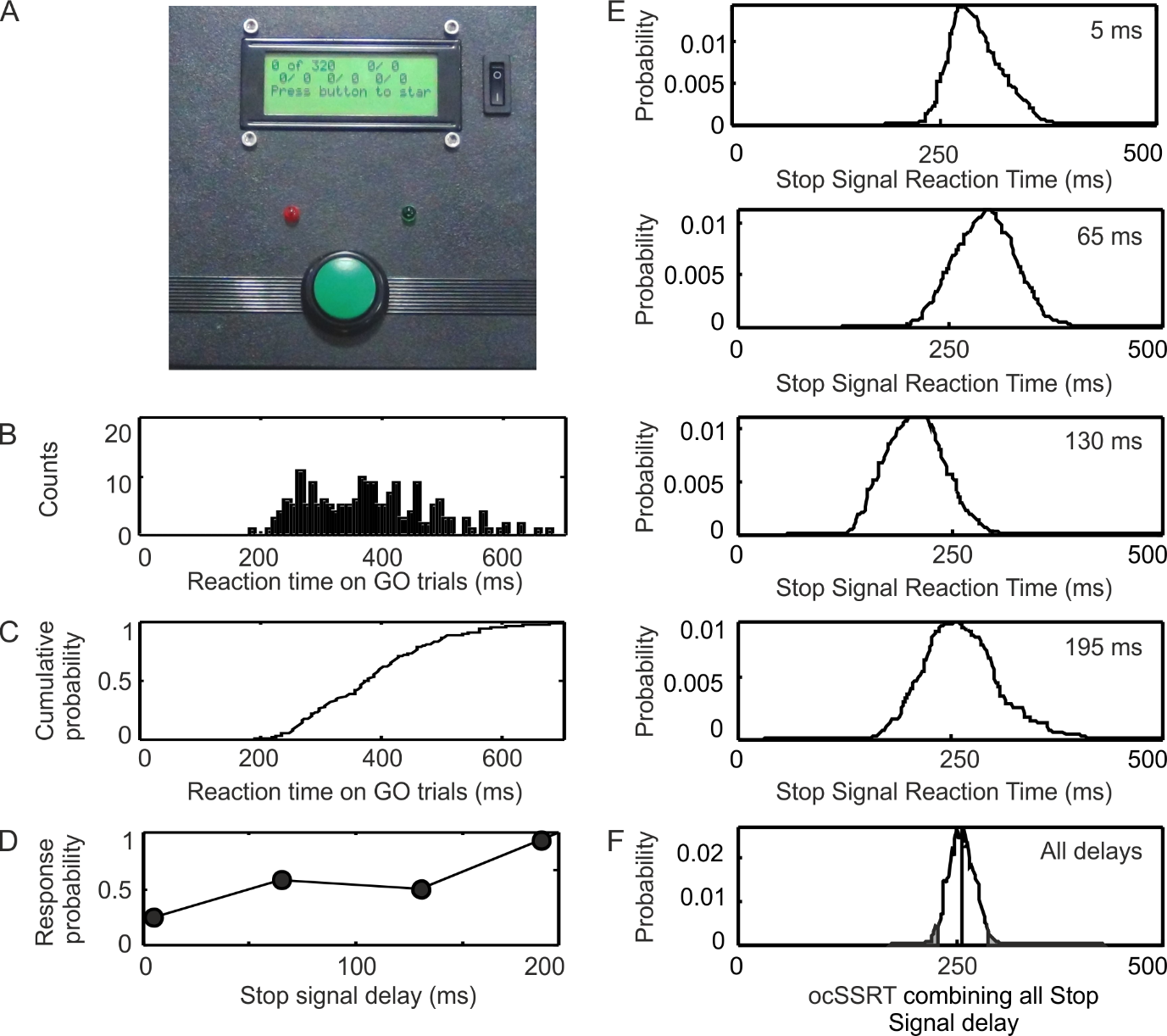


**Supplementary Figure 1. Method used to compute stop signal reaction time (SSRT)**

A, photograph of microcontroller-based box. B, histogram of reaction times determined from a single healthy subject. C, estimated cumulative probability distribution of reaction times, generated by transforming the histogram of (B). D, response probability as a function of stop signal delay. E, estimated SSRT likelihood functions computed as described in the text. The four plots show estimates derived from the four stop-signal delays used (corresponding to the four points in panel D). F, combined SSRT likelihood function, computed from the product of the four curves in (E). The mean of this distribution is shown by a central vertical line, and its 95% confidence limits as grey shading. Measures computed from a total of 256 trials: 192 GO trials (B,C) and 64 NOGO trials (16 for each delay, D,E).
